# Developmental changes in the capacity for mucosal immunoglobulin production and secretion in the intestines of growing calves

**DOI:** 10.1186/s13567-025-01648-z

**Published:** 2025-11-19

**Authors:** Yutaka Suzuki, Mutsumi Oishi, Shoko Hirota, Hideaki Hayashi, Satoshi Haga, Satoshi Koike, Yasuo Kobayashi

**Affiliations:** 1 Graduate School/Research Faculty of Agriculture, Hokkaido University, Sapporo, Japan; 2 College of Veterinary Health Nursing, Rakuno Gakuen University, Ebetsu, Japan; 3 Institute of Livestock and Grassland Science, National Agriculture and Food Research Organization (NARO), Nasushiobara, Japan; 4 Present Address: The Field Science Center for Northern Biosphere, Hokkaido University, Sapporo, Japan; 5 Present Address: Graduate School of Agricultural Science, Tohoku University, Sendai, Japan

**Keywords:** Calves, intestine, mucosal defense, immunoglobulins, development

## Abstract

Neonatal calves predominantly rely on colostral IgG for the passive transfer of immunity; however, little is known about their intrinsic capacity for mucosal immunoglobulin production and the developmental changes associated with their growth. To elucidate the developmental trajectory of mucosal immunity, we investigated changes in mucosal immunoglobulin concentrations and the expression levels of genes involved in immunoglobulin production and secretion across different growth stages in calves. The results demonstrated that fecal IgG and IgM levels exhibited transient peaks at 1 week of age according to smooth spline analysis, followed by sharp decreases, whereas IgA levels remained relatively stable and became the predominant isotype after 4 weeks. Gene expression analysis and immunohistochemistry revealed the localized expression of immunoglobulins in the intestinal mucosa, particularly IgA, which gradually increased with calf growth. The secretion of IgA is also thought to be facilitated by the upregulated expression of *PIGR*, a gene encoding the IgA transporter whose expression levels increase with calf growth. In contrast, the levels of plasma cell-recruiting chemokines and their receptors were not increased. These results suggest an important role for IgA in the mucosal defense system of the calf intestine, indicating its pivotal function in maintaining gut health following the clearance of colostral IgG.

**Supplementary Information:**

The online version contains supplementary material available at 10.1186/s13567-025-01648-z.

## Introduction

Calves are highly susceptible to epithelial-associated diseases, with morbidity and mortality rates peaking within the first month of life [1]. Diarrhea is a major contributor, constituting more than half of all unhealthy calves [2, 3]. Although colostrum is a vital source of immunoglobulins, acting locally on the intestinal mucosa and systemically through the bloodstream, its influence rapidly diminishes due to the clearance of colostral immunoglobulins [4–6]. Consequently, the immunological vulnerability of calves is partly attributed to their underdeveloped intrinsic immune function, including the intestinal mucosal defense system, during the neonatal period.

The concentration of IgG in calf serum peaks a few days after birth and subsequently undergoes a temporary decline over the first month of life, coinciding with the developmental phase of the calf's antibody-producing capacity [7–9]. This decline in serum immunoglobulin levels can increase the risk of infectious diseases, including diarrhea, in neonatal calves [9, 10]. In addition to the passive transfer of immunity from dams, the intrinsic mucosal defense system of the gut, including the production of secretory immunoglobulins, is essential for preventing pathogen infection on the gut mucosal surface through several actions, such as neutralizing virulence or impeding entry into the epithelial layer [11, 12]. Hence, proper maturation of the gut mucosal immunity in calves is crucial for preventing gut diseases, especially following the cessation of the colostral immunoglobulin effect.

Despite the importance of mucosal immunoglobulins in the gut, investigations of their developmental trajectories in calves remain limited. Previous studies have yielded inconsistent results, highlighting variations in the primary isotypes of mucosal immunoglobulin [13–16]. Other examinations at the cellular and molecular levels of gut immune system development in calves have revealed age-dependent changes in the number and distribution of gut mucosal leukocyte subpopulations [17, 18] and dynamic alterations in the transcriptome associated with mucosal innate and acquired immunity during early life [19]. Therefore, we hypothesized that the production capacity of mucosal immunoglobulins undergoes rapid development starting within several weeks after birth. However, a detailed investigation of this event, including the changes in the production of each immunoglobulin class, is lacking.

Therefore, the objective of this study was to examine the developmental dynamics of the production and secretion of each immunoglobulin class/isotype in intestinal tissues during calf growth. This involved assessing changes in immunoglobulin concentrations and elucidating the underlying cellular and molecular mechanisms.

## Materials and methods

### Animal handling and sample collection

Three animal experiments were conducted in this study. The animals in each experiment were raised and managed on different farms in the northern part of Japan. All calves were separated from their dams immediately after birth, and individual pens were managed with ad libitum access to water.

To assess immunoglobulin concentrations in feces and serum, we used five Holstein calves (one male and four females). Newborn calves received ad libitum amounts of dam colostrum (1–3 L) within 12 h post-partum via a nipple pail. From the following day, the calves were fed 2 kg of bulk milk twice daily until weaning. The starter diet (Mil-food B flake MH; Hokuren, Sapporo, Japan) and timothy hay were introduced at 1 week of age, and the calves were weaned at 6 weeks of age. Jugular venous blood and rectal feces were collected weekly from 1–12 weeks of age. Heifers and cows aged 20–49 months (*n* = 6) raised via the same management procedure on the same farm were also included as adult animals for blood and fecal samples. After coagulation, the blood samples were centrifuged at 1200 × *g* for 10 min at 4 °C to isolate the serum. Serum and fecal samples were stored at −20 °C until analysis.

Intestinal tissue samples for gene expression were adapted from a previous study [20]. To investigate the expression of immunoglobulins and the genes associated with their production and secretion in intestinal tissues, Holstein steers and heifers at 4 weeks of age (four males), 13 weeks of age (four males), and 40 weeks of age (two males and one female) were used. They received dam colostrum for the first 3 days after birth and were then raised with a commercial milk replacer (Calf Top; Zenrakuren, Tokyo, Japan). Calves euthanized at 4 weeks of age were fed only milk replacer six times daily (fed every 4 h from 0900 h). Calves euthanized at 13 and 40 weeks of age were weaned at 6 weeks of age and fed twelve meals of starter diet daily (every 2 h from 0900 h) via a continuous feeder from 4 weeks of age.

Four male Holstein calves were subjected to immunoglobulin immunohistochemistry. After birth, they were fed one bag of commercial milk replacers (Saishono-milk, Scientific Feed Laboratory, Tokyo, Japan) and pooled dam colostrum (1.5–2 L). Then, they were offered milk replacer (Calf Top EX; Zenrakuren, Tokyo, Japan). A grower feed (Zenrakuren) and chopped timothy hay were introduced at 1 and 6 weeks of age, respectively. At 12 weeks of age, the calves were euthanized for tissue sampling.

All calves were fed in accordance with the Japanese Feeding Standard for Dairy Cattle, which is designed to meet energy requirements. To collect intestinal tissue samples, euthanasia was performed by exsanguination of the carotid artery after an overdose of pentobarbital (30 mg/kg BW). Mucosal tissues were collected immediately from the duodenum, central jejunum, distal ileum, and central flexure of the ascending colon after washing with sterile saline. For gene expression analysis, the mucosal layer was collected by gentle scraping with glass slides to minimize contamination from submucosal lymphoid follicles, particularly Peyer’s patches in the ileum. The tissue samples were then snap-frozen in liquid nitrogen and stored at −80 °C until analysis.

### Measurement of immunoglobulin concentrations

The concentrations of immunoglobulins were determined via Ig class-specific ELISA, as described in our previous work [21]. Serum samples were diluted in dilution/blocking buffer (0.5% fish collagen in Tris-buffered saline supplemented with 0.05% Tween 20 (TBST), pH 7.4). For the fecal samples, frozen feces (approximately 0.5 g) were homogenized with a ninefold weight of the blocking/dilution buffer used for the serum samples, and a 1/100 volume of a protease inhibitor cocktail (Fujifilm Wako Pure Chemicals, Osaka, Japan) was added. The homogenate was centrifuged at 2000 × *g* for 5 min at 4 °C, and the supernatant was collected. The supernatant was centrifuged at 13 000 × *g* for 15 min at 4 °C to remove any residual debris. The supernatant was further diluted with blocking/dilution buffer for ELISA.

The antibodies used for IgA, IgG, and IgM measurements are listed in Additional file 1. Capturing antibodies were immobilized on ELISA plates (ELISA Plate H; Sumitomo Bakelite, Tokyo, Japan), followed by blocking with the dilution/blocking buffer described above. After washing with TBST, the standard series and diluted samples were loaded into each well, and the plates were incubated overnight at room temperature. After washing with TBST, diluted detection antibodies were added to each well, and the plates were incubated for 1 h at room temperature. After washing with TBST, a development solution (o-phenylenediamine dihydrochloride dissolved in citrate buffer) was added to each well, followed by incubation at room temperature in the dark. A stop solution (1 N HCl) was added to each well to stop color development, and the optical density at 490 nm was measured via a plate reader (1420 Multilabel Counter; PerkinElmer, Waltham, MA, USA). All standards and samples were prepared and measured in duplicate. A standard curve was generated by 4-parameter logistic curve fitting, using the nplr package in R (ver. 4.4.3). Before the assay, a dilution linearity test was performed on the serum or fecal samples. Each sample was serially diluted in blocking/dilution buffer to generate a range of concentrations, and the absorbance values were recorded. The ratio of the observed absorbance to the theoretical concentration was calculated, and linearity was confirmed if the ratio fell within the 80–120% range. A spike recovery test was also conducted to evaluate assay accuracy. The baseline concentrations of the target analytes were measured, and known concentrations of the analytes were spiked into the samples. The recovery percentage was calculated by comparing the measured concentration to the expected value. The assay was considered accurate if the recovery rate was within 80–120%, a range chosen owing to the large matrix effect in fecal samples.

### Gene expression analysis

Frozen tissue samples were crushed into a fine powder using RNAiso Plus (Takara Bio, Shiga, Japan) in liquid nitrogen and thawed at room temperature to obtain homogenates. The homogenates were mixed with chloroform by vortexing and centrifuged at 12 000 × *g* for 15 min at 4 °C for phase separation. The aqueous phase was collected in a new tube and mixed with equal volumes of 70% ethanol. This mixture was loaded onto a silica-based purification column (Favourgen, Ping Tung, Taiwan) to purify the total RNA following the manufacturer’s protocol. The purity and integrity of the RNA samples were checked using a NanoDrop 2000 spectrophotometer (Thermo Fisher Scientific, Waltham, MA, USA) and agarose gel electrophoresis, respectively.

The expression levels of genes encoding the heavy chains of each immunoglobulin isotype and genes associated with the production and secretion of immunoglobulins were assessed via qRT‒PCR. First-strand cDNA was synthesized from 500 ng of total RNA via ReverTra Ace qPCR RT Master Mix (Toyobo, Osaka, Japan). A KAPA SYBR Fast qPCR Kit (Kapa Biosystems, Wilmington, MA, USA) and a LightCycler 480 (Roche, Basel, Switzerland) were used to perform real-time PCR. The primer sequences are listed in Additional file 2. The PCR amplification efficiency for each primer pair, which is theoretically 100% (= 1.0), was checked by constructing standard curves via five-point serial dilutions of pooled cDNA from intestinal tissues. Relative quantification of gene expression levels was performed via the ΔΔCt method [22]. The geometric means of the Ct values of β-actin (*ACTB*), ribosomal protein S9 (*RPS9*), and glyceraldehyde-3-phosphate dehydrogenase (*GAPDH*) were used for normalization of the target genes [23]. Gene expression data were further transformed into z scores for heatmap visualization and statistical analysis.

### Immunohistochemistry

For immunostaining, fresh frozen tissues from the duodenum, jejunum, ileum, and colon were sectioned to 12 μm thicknesses and fixed with ice-cold acetone for 15 min. The tissue sections were blocked with buffer containing 1% IgG-free bovine serum albumin (Wako Pure Chemical, Osaka, Japan) and 10% normal sheep serum in TBST for 30 min. The sections were incubated overnight at 4 °C with FITC-conjugated anti-bovine IgA, IgG, or IgM antibodies diluted in the same buffer used for blocking. The antibodies used for immunohistochemistry are listed in Additional file 3. The specificity of the antibodies was checked by staining sections with normal sheep IgG, which served as an isotype control (Additional file 4). The cell nuclei were counterstained with Hoechst 33,258. After being washed with TBST, the sections were sealed with mounting medium and coverslips and observed under a BZ-9000 fluorescence microscope (Keyence, Osaka, Japan).

### Statistical analysis

All the statistical analyses were conducted in R (version 4.4.3). Descriptive statistics for the data obtained in each experiment, corresponding to Figures 2, 3, 5, 6 and 7, are summarized using *summarise* function and shown in Additional files 5–9. To assess temporal dynamics in immunoglobulin concentrations and tissue-specific differences in related gene expression, generalized additive mixed models (GAMMs) were applied depending on the experimental design [24]. The overall modelling strategy was structured as follows.

**Figure 1:**
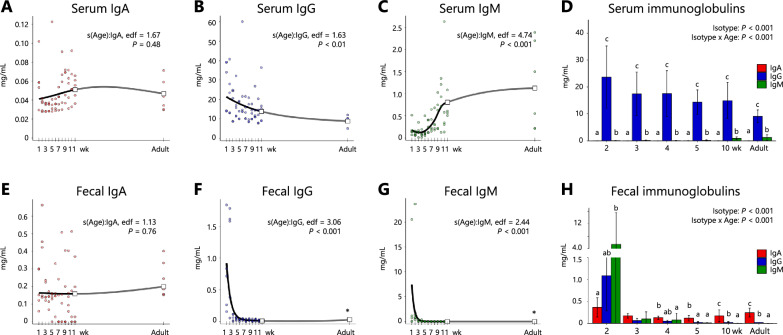
**Age-associated changes in immunoglobulin concentrations in calf serum and feces**. The observed values for IgA, IgG, and IgM are plotted for serum (**A**–**C**) and feces (**E**–**G**) from calves at 1 to 12 weeks of age and in adult cattle, together with fitted smooth spline trajectories derived from generalized additive mixed models (GAMMs). Data for 12-week-old adults are indicated by open squares and were additionally compared using Holm-adjusted pairwise tests; asterisks indicate statistical significance (*P* < 0.05) for these comparisons. Isotype-specific comparisons are shown for serum (**D**) and feces (**H**) at selected time points (2, 3, 4, 5, 10 weeks, and adult), with multiplicity adjusted by the multivariate t method. Different alphabets indicate statistical significance (*P* < 0.05) between isotypes within each age.

To evaluate longitudinal changes in serum and fecal immunoglobulin concentrations (IgA, IgG, and IgM), we fitted GAMMs using the *mgcv::gam* function (Figure 1). All available weekly data from 1 to 12 weeks of age were included, together with additional samples from adult animals (mean age 49 weeks). The immunoglobulin concentration (*Conc*) was modelled with a gamma distribution (log link) to accommodate the right-skewed nature of the data. Fixed effects included immunoglobulin isotype (*Isotype*) and a smooth term of calf age, with isotype-specific smooths specified as *s(Age, by* = *Isotype)*. Individual calves were modelled as a random effect using *s(Animal, bs* = *“re”*). The full model formula was as follows: *Conc* ~ *Isotype* + *s(Age, by* = *Isotype)* + *s(Animal, bs* = *“re”)*. The significance of the fixed effect and smooth terms for each isotype was assessed using likelihood ratio test and approximate F tests, respectively. In addition to assessing the significance of the smooth term of calf age, we conducted post hoc pairwise contrasts between 12-week-old individuals and adults for each isotype using estimated marginal means via the *emmeans* package to address the absence of observations within this interval. *P* values were adjusted using the Holm method across isotypes. Furthermore, pairwise comparisons among isotypes were performed at each observed week using the *emmeans* package, with *P* values adjusted by the multivariate t (*mvt*) method, and the results were summarized with *multcomp:cld* function.

**Figure 2:**
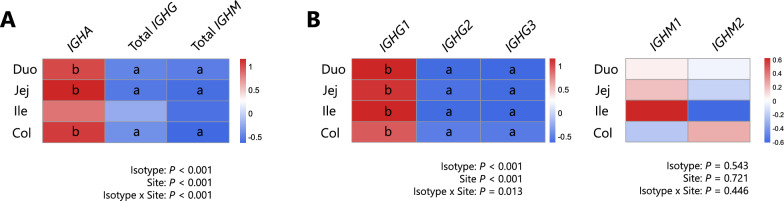
**Comparison of the expression levels of immunoglobulins among classes and subclasses.** The expression levels of genes encoding the heavy chain of immunoglobulin were compared between classes (**A**) or subclasses (**B**) in each site of the intestine of calves at 40 weeks of age. Total *IGHG* and *IGHM* stand for the summed expression levels of subclasses of each immunoglobulin class. Different letters indicate statistical significance (*P* < 0.05) between immunoglobulin classes (A) or subclasses (B) within each site of the intestine according to pairwise comparisons adjusted via the multivariate t method, preceded by generalized additive mixed model analyses. Duo: duodenum, Jej: jejunum, Ile: ileum, and Col: colon.

**Figure 3:**
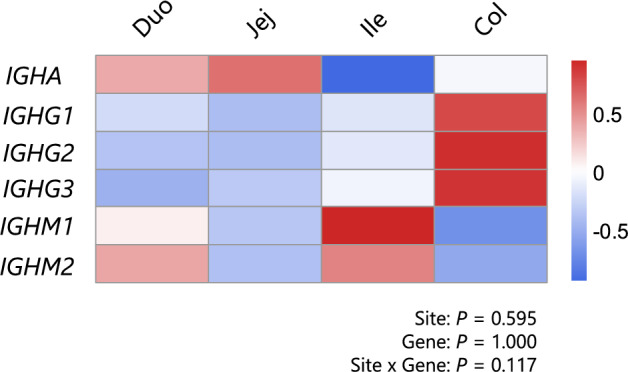
**Comparison of the expression levels of immunoglobulins among sites in the intestine**. The expression levels of each gene encoding the heavy chain of immunoglobulins were compared across the sites of the intestine from calves at 40 weeks of age. Statistical analysis was performed using a generalized additive mixed model analysis to determine the effects of tissue site, gene and their interaction. Duo: duodenum, Jej: jejunum, Ile: ileum, and Col: colon.

To compare gene expression profiles across immunoglobulin classes (*IGHA*, total *IGHG*, and total *IGHM*) and subclasses (*IGHG1–3*, and *IGHM1–2*), GAMMs were constructed using z scored gene expression data. The models incorporated anatomical sites of the intestine (*Site*) and immunoglobulin class/subclass (*Isotype*) as fixed effects, along with their interaction. Individual calves were included as a random effect using *s(Animal, bs* = *“re”*). The model structure was as follows: *z score* ~ Site * Isotype + *s(Animal, bs* = *“re”)*. All the models were fitted using the scaled t distribution (*scat()* family) to ensure robustness to deviations from normality. Post hoc comparisons of gene expression were performed within sites across the immunoglobulin class and subclasses (Figure 2) and within each isotype across tissue sites at the selected age (Figure 3) *emmeans* with the *mvt* adjustment and *cld* function as described above.

To assess age-related changes in gene expression (Figures 5 and 6), GAMMs were applied separately to z scored expression data for each gene. The models included *Site*, *Age* (treated as a categorical factor), and their interaction as fixed effects, with individual calves modelled as a random effect using *s(Animal, bs* = *“re”)*. All the models were fitted using the scaled t-distribution family (*scat()* in mgcv) to account for potential deviations from normality. The models were as follows: *z score* ~ Site * Age + *s(Animal, bs* = *“re”)*. For Figure 5, statistical comparisons were performed within each immunoglobulin gene to evaluate age-dependent differences in expression across intestinal sites. For Figure 6, similar models were used to assess age-related changes in genes involved in immunoglobulin production and transport. In both cases, post hoc comparisons were conducted using the *emmeans* package, with pairwise contrasts between age groups performed within each site. *P* values were adjusted for multiple testing using the *mvt* method, and the results were summarized using the *cld* function.

For the analysis in Figure 7, a GAMM was fitted to z scored expression data of immunoglobulin-related genes with *site, gene*, and their interaction as fixed effects. The model structure was as follows: *z score* ~ Site * Gene + *s(Animal, bs* = *“re”)*, also using the *scat()* family. Post hoc comparisons of site-specific expression within each gene were performed as described above.

Even when the interaction between fixed effects was not statistically significant, site–wise or gene–wise post hoc comparisons were conducted to test biologically driven hypotheses regarding site– or gene–specific expression patterns. Multiple comparison adjustments were applied as described above.

## Results

### Concentrations of immunoglobulins in the serum and feces

To characterize post-natal changes in the immunoglobulin profiles of calves, GAMMs were used to analyse the serum and fecal concentrations of IgA, IgG, and IgM (Figures 1A–H). In the serum, the smooth terms of age were statistically significant for IgG (edf = 1.63, *P* < 0.01) and IgM (edf = 4.74, *P* < 0.001), whereas IgA had no significant effect (Figures 1A–C). The fitted trajectories indicated a gradual decline in IgG concentrations during the juvenile period, with mean values decreasing from 28.1 ± 13.0 mg/mL at 1 week to 17.3 ± 11.3 mg/mL at 12 weeks and reaching 9.1 ± 2.4 mg/mL in adults. IgM showed a nonlinear increase from 0.084 ± 0.068 mg/mL at 4 weeks to a peak of 0.86 ± 0.48 mg/mL at 10 weeks, followed by stabilization. In contrast, the IgA concentrations remained relatively constant (0.060 ± 0.026 mg/mL at 1 week) without significant temporal variation. Planned contrasts between 12-week-old patients and adults revealed no significant differences in IgA, IgG or IgM, which was consistent with the trajectories (Figures 1A-C). Pairwise isotype comparisons at selected time points indicated that the level of IgG was consistently greater than that of IgA and IgM at 2, 3, 4, 5, and 10 weeks, as well as in adults (Figure 1D; 681– and 263–fold greater at 2 weeks, and 203– and eightfold greater in adults, respectively), confirming its predominance in serum.

In the fecal samples, smooth terms were significant for IgG (edf = 3.06, *P* < 0.001) and IgM (edf = 2.44, *P* < 0.001), whereas IgA had no significant effect (Figures 1E–G). The fitted trajectories showed transient peaks for IgG and IgM at 1 week (0.76 ± 0.67 mg/mL and 4.49 ± 8.99 mg/mL, respectively), followed by sharp declines to persist at low levels. Fecal IgA remained relatively stable (0.1–0.2 mg/mL). Planned contrasts between 12-week-old adults and control adults revealed a modest but significant increase in IgG (ratio = 3.19) and a decrease in IgM (ratio = 0.0427), whereas IgA remained stable (Figures 1E-G). However, these differences were minor compared with the substantial early-life declines observed for IgG and IgM. Pairwise comparisons among isotypes confirmed that IgG and IgM predominated during the first 2 weeks, but IgA levels exceeded both from 4 weeks onwards and remained the dominant mucosal isotype into adulthood (Figure 1H).

### Comparison of the expression levels of immunoglobulins among isotypes

To characterize the developmental changes in the gene expression of the immunoglobulin class and subclass in the intestinal mucosal tissue, we analysed data from calves at 40 weeks of age when the capacity for immunoglobulin production was thought to be sufficient, employing GAMMs fitted with the *scat* family. When the immunoglobulin classes (*IGHA*, total *IGHG* and total *IGHM*; Figure 2A) were compared, significant effects on the isotype (*P* < 0.001) and intestinal site (*P* < 0.001) as well as their interaction (*P* < 0.001) were detected. Post hoc comparisons within each site revealed that *IGHA* expression in the duodenum, jejunum and colon was significantly greater than that of total *IGHG* and total *IGHM*. In comparisons of immunoglobulin subclasses (*IGHG1–3* and *IGHM1–2* in Figure 2B), the fixed effects of isotype and site, as well as their interaction, were statistically significant (*Isotype*: *P* < 0.001; *Site*: *P* < 0.001; interaction: *P* < 0.05). However, no significant differences were detected among the *IGHM* subclasses. Post hoc comparisons indicated that *IGHG1* expression was significantly higher than that of *IGHG2* and *IGHG3* across all intestinal sites. The *IGHM1* and *IGHM2* expression levels did not differ significantly among the sites.

### Expression and localization of immunoglobulins in sites of the intestine

No statistically significant effects were observed for the fixed terms or the interaction in the GAMM used to evaluate the expression patterns of immunoglobulin isotypes among anatomical sites of the intestine, indicating comparable expression levels of each isotype across the intestinal sites (Figure 3).

**Figure 4:**
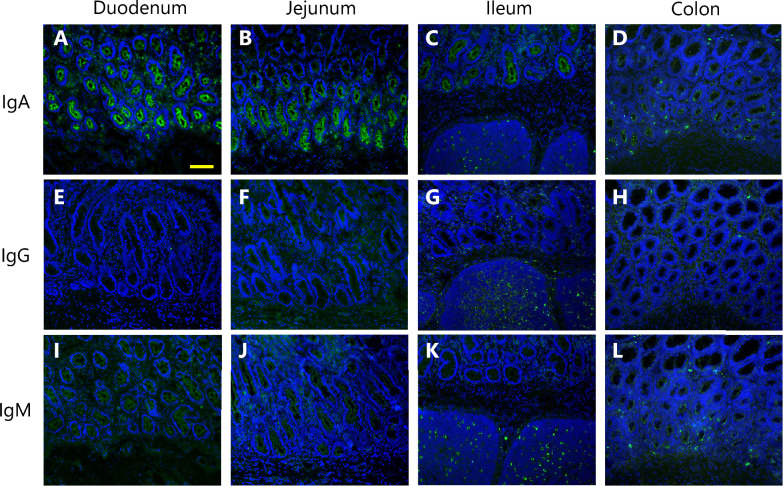
Immunohistochemistry of immunoglobulins in the intestinal tissues of calves. IgA (**A–D**), IgG (**E–H**), and IgM (**I–L**) were visualized by fluorescence immunohistochemistry (green) in intestinal tissue sections from calves at 12 weeks of age (representative images are shown). The cell nuclei were counterstained (blue). The yellow scale bar indicates 100 μm.

Fluorescence immunohistochemistry of intestinal tissues demonstrated the presence of IgA, IgG, and IgM proteins in the epithelial tissues of each intestinal site. In particular, IgA and IgM were detected on the mucosal surfaces of all the sites of the intestine (Figures 4A–D and I–L), whereas IgG was more likely to be present in the lamina propria, especially in the colon (Figures 4E–H). In the ileum, IgG- and IgM-producing cells were abundant in the lymphoid follicles (Figures 4G and K), whereas IgA-producing cells were less abundant (Figure 4C).

### Age-associated changes in the expression levels of immunoglobulins in the intestine

**Figure 5:**
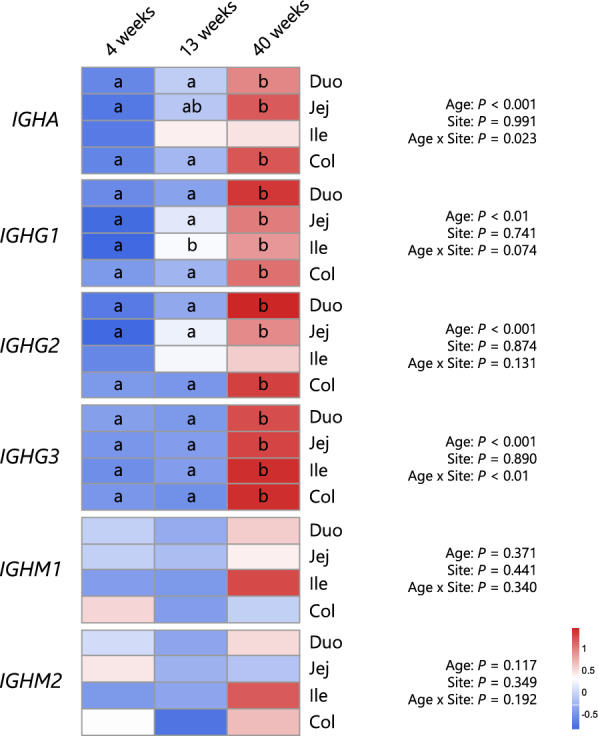
**Age-associated changes in the expression levels of immunoglobulins in intestinal sites**. The expression levels of genes encoding the heavy chain of immunoglobulin in intestinal tissues were compared between calves of different ages. Different letters indicate statistical significance (*P* < 0.05) between ages within each site of the intestine according to pairwise comparisons adjusted using the multivariate t method, followed by generalized additive mixed model analyses. Duo: duodenum, Jej: jejunum, Ile: ileum, and Col: colon.

To examine age-associated changes in immunoglobulin gene expression, GAMMs were fitted separately for each gene using the *scat* family, with age and intestinal tissue site as fixed effects and individual calves as random effects (Figure 5). For genes encoding IgA (*IGHA*), IgG subclasses (*IGHG1–3*), and IgM subclasses (*IGHM1–2*), significant effects of age, site, and their interaction were observed, depending on the gene. For *IGHA*, significant effects were detected for site (*P* < 0.001) and the interaction between age and site (*P* < 0.05). Post hoc comparisons revealed that *IGHA* expression levels in the duodenum, jejunum and colon increased with age, with the highest expression levels occurring at 40 weeks of age. For *IGHG1*, the effect of age (*P* < 0.01) was significant. Post hoc analysis indicated that *IGHG1* expression levels were higher at 40 weeks of age than at 4 and 13 weeks of age in all intestinal sites except the ileum, where expression was already elevated at 13 weeks. For *IGHG2*, a significant effect of age (*P* < 0.001) was observed, with increased expression at 40 weeks detected in the duodenum, jejunum, and ileum. Similarly, for *IGHG3*, significant effects of age (*P* < 0.001) and the age × site interaction (*P* < 0.01) were detected, with expression levels increasing markedly at all sites between 13 and 40 weeks of age. For the IgM subclasses, no significant effects were detected in the GAMMs for *IGHM1* and *IGHM2*, including the main effects of site and age or their interaction.

### Age-associated changes in the expression levels of genes associated with immunoglobulin production and secretion in the intestine

**Figure 6:**
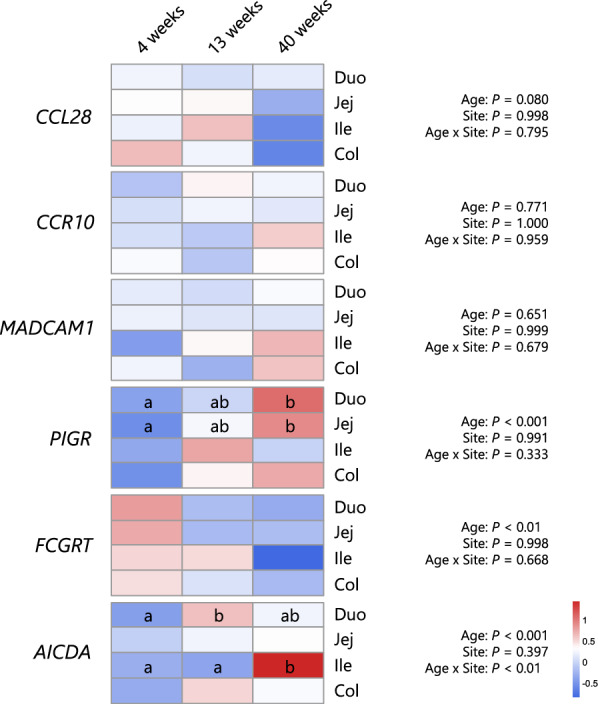
**Age-associated changes in the expression levels of genes involved in immunoglobulin production and secretion at sites in the intestine.** The expression levels of genes associated with immunoglobulin production and secretion in intestinal tissues were compared between calves of different ages. Different alphabets indicate statistical significance (*P* < 0.05) between ages within each site of the intestine according to pairwise comparisons adjusted using the multivariate t method, preceded by generalized additive mixed model analyses. Duo: duodenum, Jej: jejunum, Ile: ileum, and Col: colon.

To examine age-associated changes in genes involved in immunoglobulin production and luminal secretion, we analysed the expression of six representative genes via GAVM with the *scat* family (Figure 6). No statistically significant effects of age, site, or their interaction were detected for *CCL28*, *CCR10*, or *MADCAM1* (*P* > 0.05 for all terms), and post hoc comparisons revealed no significant differences across calf ages. In contrast, *PIGR* expression was significantly affected by age (*P* < 0.001). Post hoc analysis indicated gradual increases in the expression levels of IgA transporters in the duodenum and jejunum with calf growth, suggesting age-related enhancement of epithelial IgA transport capacity in these proximal intestinal segments. *FCGRT*, which encodes the neonatal Fc receptor (FcRn) responsible for IgG transcytosis, also exhibited a significant effect on age (*P* < 0.01). However, post hoc comparisons did not reveal statistically significant pairwise differences between time points within any site. *AICDA*, a key regulator of somatic hypermutation and class switch recombination, was significantly affected by age (*P* < 0.001) and the age × site interaction (*P* < 0.01). Compared with that at earlier time points, expression in the ileum was markedly upregulated at 40 weeks of age (*P* < 0.05). In the duodenum, expression at 13 weeks was significantly greater than that at 4 weeks. No significant age-related changes were observed in the jejunum or colon.

### Comparison of the expression levels of genes associated with immunoglobulin production and secretion among sites of the intestine

**Figure 7:**
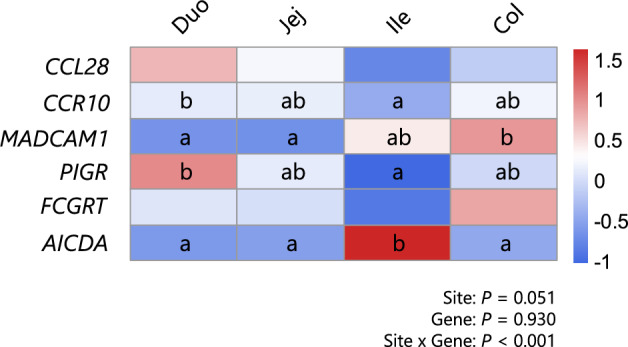
**Comparison of the expression levels of genes involved in immunoglobulin production and secretion among the sites of the intestine.** The expression levels of genes associated with immunoglobulin production and secretion were compared across the intestine of calves at 40 weeks of age. Different letters indicate statistical significance (*P* < 0.05) between the sites of the intestine within each gene according to pairwise comparisons adjusted using the multivariate t method, preceded by a generalized additive mixed model analysis. Duo: duodenum, Jej: jejunum, Ile: ileum, and Col: colon.

A GAMM was fitted with the *scat* family to evaluate the spatial expression patterns of immunoglobulin-related genes across intestinal sites (Figure 7). A significant interaction between site and gene (*P* < 0.001) was detected, although the main effects of site (*P* = 0.051) and gene (*P* = 0.930) were not individually significant. Post hoc comparisons revealed gene-specific regional patterns. For *CCL28* and *FCGRT*, no significant differences in expression levels were observed among the sites. *CCR10* expression in the duodenum was significantly greater than that in the ileum, with intermediate levels in the jejunum and colon. *MADCAM1* expression was significantly greater in the colon than in the duodenum and jejunum. The expression of PIGR in the duodenum was significantly greater than that in the ileum, whereas intermediate values were detected in the colon and jejunum. The expression of AICDA in the ileum was significantly greater than that in the duodenum, jejunum, or colon.

## Discussion

Immunoglobulins, particularly IgG, have been studied extensively in relation to colostrum feeding in calves [25]. Colostral IgG is indispensable for the defense system of calves, preventing them from prevalent diseases such as diarrhea, with a peak incidence during the first few months of life [1–3]. Intestinal resecretion of colostral IgG absorbed in calf blood is considered the major route of IgG clearance in calves, accounting for approximately 70% of the clearance, although it is active shortly during the neonatal period [4]. Despite the short-term effect of colostral IgG, insights into the intrinsic capacity of calves to produce mucosal immunoglobulins, which are potentially essential for the development of gut epithelial defense after the clearance of colostral IgG, have been limited. Although some prior investigations have revealed the presence and dynamics of lymphoid and myeloid cell populations in the gut epithelium [14, 17], changes in the actual concentration of each mucosal immunoglobulin class and the underlying mechanisms remain unclear.

Our study included calves with serum IgG concentrations comparable to those reported in previous research [26–28] (Figure 1B), suggesting successful acquisition of colostral IgG and proper development of systemic immunity. According to the GAMM smooth spline analysis, the serum IgG and IgM levels displayed significant age-related trajectories, whereas the serum IgA level did not (Figures 1A–C). Furthermore, isotype-specific comparisons at selected time points revealed that serum IgG was consistently more abundant than IgA and IgM were (Figure 1D), which aligns with findings from previous studies that demonstrated sustained IgG dominance in calves [29].

In the feces, IgG and IgM levels peaked at approximately 1 week of age according to the spline curves and declined sharply thereafter (Figures 1E–F). Fecal IgG and IgM concentrations did not reflect changes in serum levels (Figures 1B–C). Although calves are believed to initiate endogenous IgG synthesis between 36 h and 3 weeks of age [30, 31], the prominence of colostral IgG and IgM excretion during this early window likely drove the observed peaks.

In our study, fecal IgA concentrations surpassed serum levels (Figures 1A and E), reflecting the prominent production capacity of IgA in the intestinal mucosa rather than its transfer from the blood, as is known in other mammals [32] Notably, up to 2 weeks of age, fecal IgG and IgM were predominant, but thereafter, fecal IgA concentrations remained relatively stable and became consistently higher than those of IgG and IgM after 4 weeks of age—a transition clearly illustrated by the spline curves and isotype-specific comparisons (Figures 1E‑H). This pattern highlights that prominent post-natal immunological maturation might occur at the intestinal mucosal level. Although the gut mucosal immune system in calves dynamically develops from the prepartum period to the post-natal period, changing the cellular populations, including T, B, and antigen-presenting cells in mucosal lymphoid follicles [14, 33], previous studies have yielded inconsistent results regarding the primary mucosal immunoglobulin class or isotype found in the calf gut. IgG1 was predominant in the gut lumen of calves at 2–3 weeks of age, with IgA present at a much lower proportion, whereas cattle at 18 months of age presented an elevated proportion of IgA, with IgG1 still being the most abundant mucosal immunoglobulin [15]. However, a more recent study employing a larger number of mature beef cattle demonstrated a higher fecal concentration of IgA than that of IgG and IgM, which is consistent with our results [16]. Although colostral IgG in calf blood undergoes gut luminal excretion, indicating its potential contribution to mucosal defense against luminal pathogens, its half-life is estimated to be up to 16 days [4, 34, 35]. Therefore, our data suggest that IgA is the primary gut mucosal immunoglobulin in young calves, with a more significant contribution to epithelial defence, particularly after approximately 4 weeks of age.

To assess the local production capacity of immunoglobulins in the intestinal mucosa, we investigated the expression of genes encoding the heavy chain of each immunoglobulin isotype in relatively mature cattle (40 weeks of age). *IGHA* expression exceeded that of total *IGHG* and *IGHM* in the duodenum, jejunum, and colon (Figure 2). Immunohistochemistry demonstrated that IgA was abundant on the mucosal surface across the intestinal sites (Figure 4). These findings highlight the greater potential of the intestinal mucosal tissue to produce IgA than IgG and IgM and support the difference in the fecal concentrations of each immunoglobulin discussed above. Furthermore, our data revealed substantial *IGHA* expression in the colonic mucosa, comparable to that observed in the small intestine (Figures 3 and 4), suggesting a significant contribution of the colon to IgA production. In terms of immunoglobulin subclasses, *IGHG1* presented higher expression levels than did *IGHG2* and *IGHG3*, whereas no differences were detected when intestinal sites were compared (Figures 2 and 3). This might reflect the fact that IgG1 is known as the predominant secretory form of IgG, particularly in cow colostrum [36, 37], although further investigations should be performed to identify the determinants of this mucosal IgG subclass balance, considering their differing roles in cytotoxicity, phagocytosis and inflammatory responses [38]. In contrast, IgM did not show clear trends in terms of expression differences between subclasses (Figure 2), but the IgM protein was clearly observed in the ileal lymphoid follicles (Figure 4), indicating that the ileum in cattle serves as a source of B cells that subsequently undergo maturation and class switching [39, 40]. Additionally, total *IGHM* expression in the ileum was lower than that in the *IGHA*, although the difference was not statistically significant. The *IGHM1* and *IGHM2* expression levels were also comparable to those observed in other intestinal sites, suggesting that contamination by submucosal lymphoid follicles was likely limited. These findings indicate that gene expression analysis primarily reflects activity at the mucosal surface.

To understand the developmental trajectory of the local production of immunoglobulins on the gut mucosa, we compared the expression of *IGH* genes in calves of different ages (Figure 5). *IGHA* expression markedly increased in the duodenum, jejunum and colon at 40 weeks of age, although it was detectable at 4 weeks of age. A previous study demonstrated detectable expression levels of the gene encoding the J-chain of secretory immunoglobulins in the small intestine of calves, with upregulation occurring at 0–21 days after birth [19]. Additionally, the development of jejunal and ileal Peyer’s patches is initiated by post-partum exposure to commensal microbes, with rapid growth of these tissues over one month of age [14]. Hence, it has been suggested that gut IgA production is initiated in the neonatal period and continues through the growth phase, which extends to approximately 40 weeks of age in calves. *IGHG1*, *IGHG2*, and *IGHG3* presented relatively high expression levels in intestinal tissues at 40 weeks of age. However, their fecal concentrations showed different trends, with decreasing concentrations in older animals. This can be attributed to the clearance of colostral immunoglobulins from the gut lumen, as previously discussed. Consequently, IgA was deemed to have a more substantial effect on the development of gut immunity, particularly after 4 weeks of age, considering its changes in expression and higher luminal concentrations in growing calves.

The production and secretion of immunoglobulins are mediated by various processes, including B-cell maturation, recruitment and the transport of immunoglobulin molecules across gut epithelial cells [41, 42]. Therefore, to further investigate the developmental mechanisms of mucosal immunoglobulins, we assessed the expression levels of factors associated with this mechanism (Figs. 6 and 7). Specific chemokines and their receptors play critical roles in recruiting B cells from the circulation to peripheral tissues, including the gut mucosa. The CCL28-CCR10 pathway is a primary signalling pathway that recruits IgA-producing plasma cells, which are highly expressed in bovine epithelial tissues, including the small intestine [43, 44]. In our study, their expression levels did not significantly change with age, although *IGHA* levels increased with calf growth. *MADCAM1*, a cell-surface adhesion protein involved in plasma cell recruitment to the intestinal mucosa [45], also did not show age-associated changes in its expression levels. Because the molecules that induce plasma cell recruitment do not show developmental changes, the mode of upregulation of intestinal immunoglobulin genes is not fully understood from the present results. Further studies are needed to determine the factors responsible for the accumulation and maintenance of plasma cells, such as APRIL or other molecules [46]. *CCR10* and *MADCAM1* showed site-associated differences. Lower CCR10 expression was observed in the ileum than in the ileum, suggesting that intestinal sites other than the ileum may serve as more likely targets for the homing of IgA-producing cells, although comparisons of *IGHA* expression between sites were not statistically significant (Figure 3). Similarly, a higher *MADCAM1* expression level was observed in the colon, with no significant difference in *IGHA* expression between tissue sites. These observations highlight the need for more detailed investigations in future studies.

Immunoglobulins are transported across gut epithelial cells via receptor-mediated transcytosis. PIgR, which is expressed in intestinal epithelial cells, is responsible for the secretion of dimeric IgA and polymeric IgM molecules [47, 48]. In our study, the gene expression levels of *PIGR* were greater in the duodenum than in the ileum, with intermediate levels in the jejunum and colon, and its expression in the duodenum and jejunum significantly increased with calf growth. These results suggest a greater secretory potential of IgA in these intestinal sites, which may explain the elevated proportion of IgA in feces with calf growth. FcRn, encoded by *FCGRT*, is the fetal form of the IgG receptor responsible for the bidirectional transport of IgG across the epithelial layer and its recycling [49, 50]. In our study, the expression level of this gene did not differ between the intestinal sites. Although multiple comparisons failed to reveal a developmental change, the effect of calf age was significant, with decreased expression levels at 40 weeks of age, supporting the finding that fecal IgG concentrations remained low after 3 weeks of age (Figure 1). Activation-induced cytidine deaminase (AICDA), an enzyme involved in repertoire maturation and class switching of immunoglobulins [51, 52], is reported to be highly expressed in Peyer’s patches of the bovine ileum [53]. Our results revealed that the highest expression of *AICDA* in the ileum increased with calf growth. Considering the low expression level of *PIGR* in the ileum, this intestinal region in calves is more likely to serve as a site for B-cell proliferation and maturation within its lymphoid follicles, consequently contributing to immunoglobulin secretion in the duodenum, jejunum, and colon [39].

In summary, our results demonstrated that IgA became the primary isotype of mucosal immunoglobulins after 4 weeks of age, whereas the levels of fecal IgG and IgM peaked at 1 week and declined rapidly thereafter. These findings suggest that a rapid shift in the balance of immunoglobulin isotypes occurs during this period, which is attributable to changes in the colostrum-derived supply and the maturation of intrinsic immunoglobulin production. Gene expression assessment revealed substantial local production of IgA in the intestine, which gradually increased with calf growth. The secretion of IgA is also thought to be promoted by the increased expression of *PIGR,* which is significantly greater at 40 weeks of age. The ileum is thought to be the source of functional B cells, which subsequently contribute to mucosal immunoglobulin production. Moreover, the small sample size was a major limitation of this study, potentially affecting both the detection of subtle molecular changes regarding plasma cell recruitment and maturation and the robustness of spline fitting, particularly for the limited adult group. In conclusion, the present study provides insights into intestinal immunity development, to which the upregulation of IgA contributes significantly, although the precise mechanisms—especially the recruitment and differentiation of IgA–producing plasma cells—remain to be elucidated in a future study with a larger sample size. Our results suggest a pivotal role for IgA in maintaining the gut health of growing calves.

## Supplementary Information

**Additional file 1. Antibodies used for ELISA**.

**Additional file 2. PCR primers used for qRT‒PCR**.

**Additional file 3. Antibodies used for immunohistochemistry**.

**Additional file 4. Isotype control of immunohistochemistry of intestinal tissues from calves**. Intestinal sections from calves at 12 weeks of age were reacted with normal sheep IgG conjugated with FITC (*n* = 4: representative pictures are shown). Nuclei were counterstained (blue). The yellow scale bar indicates 100 μm.

**Additional file 5. Descriptive statistics for the data corresponding to Figure 2.**

**Additional file 6. Descriptive statistics for the data corresponding to Figure 3.**

**Additional file 7. Descriptive statistics for the data corresponding to Figure 5.**

**Additional file 8. Descriptive statistics for the data corresponding to Figure 6.**

**Additional file 9. Descriptive statistics for the data corresponding to Figure 7.**

## Data Availability

The raw data supporting the conclusions of this article will be made available from the corresponding author upon reasonable request.
