# Supplementary material for: Developmental changes in the capacity for mucosal immunoglobulin production and secretion in the intestines of growing calves
Source: Vet Res. 2025 Nov 19;56:220. doi: 10.1186/s13567-025-01648-z (PMC12628562; doi:10.1186/s13567-025-01648-z)
Supplement: Supplementary file 1 — Additional file 1. Antibodies used for ELISA. [file 13567_2025_1648_MOESM1_ESM.docx]

| Target | Standards | Capturing antibody | | Detecting antibody | |
| --- | --- | --- | --- | --- | --- |
|  |  | Product information | Dilution ratio | Product information | Dilution ratio |
| Bovine IgA | Reference serum | Fortis life science  A10-131A | For serum sample, 1:10 000 | Fortis life science  A10-131P | For serum sample, 1:50 000 |
|  |  |  | For fecal sample, 1:5000 |  | For fecal sample, 1:25 000 |
| Bovine IgG | Fortis life science  P10-115 | Fortis life science  A10-118A | For serum sample, 1:2000 | Fortis life science  A10-118P | For serum sample, 1:100 000 |
|  |  |  | For fecal sample, 1:2000 |  | For fecal sample, 1:100 000 |
| Bovine IgM | Rockland  001-0107 | Fortis life science  A10-101A | For serum sample, 1:3000 | Fortis life science  A10-101P | For serum sample, 1:25 000 |
|  |  |  | For fecal sample, 1:3000 |  | For fecal sample, 1:25 000 |
