# Supplementary material for: Developmental changes in the capacity for mucosal immunoglobulin production and secretion in the intestines of growing calves
Source: Vet Res. 2025 Nov 19;56:220. doi: 10.1186/s13567-025-01648-z (PMC12628562; doi:10.1186/s13567-025-01648-z)
Supplement: Supplementary file 2 — Additional file 2. PCR primers used for qRT‒PCR. [file 13567_2025_1648_MOESM2_ESM.pdf]

| Gene name | Accession No. | | Sequences (5' - 3') | Length (bp) | Efficiency* |
|---|---|---|---|---|---|
| Actin beta *(ACTB)* | NM_173979.3 | F | GAATCCTGCGGCATTCACGA | 192 | 0.94 |
| | | R | AGGGGGCGCGATGATCTTG | | |
| Ribosomal protein S9 *(RPS9)* | NM_001101152.2 | F | CAAAACCTATGTGACCCCGC | 75 | 0.96 |
| | | R | TACTCGCCGATCAGCTTCAG | | |
| Glyceraldehyde-3-phosphate dehydrogenase *(GAPDH)* | NM_001034034.2 | F | CCTGGAGAAACCTGCCAAGT | 214 | 1.02 |
| | | R | GCCAAATTCATTGTCGTACCA | | |
| Immunoglobulin heavy constant alpha *(IGHA)* | AF109167 | F | CTGCCCCGCGAGAAGTAT | 238 | 1.01 |
| | | R | CATCCACCTCTGACATGACCA | | |
| Immunoglobulin heavy constant gamma 1 *(IGHG1)* | X62916 | F | GGACACCCTCACAATCTCGG | 121 | 1.01 |
| | | R | CGTGGCTGTGTTTACCTCCA | | |
| Immunoglobulin heavy constant gamma 2 *(IGHG2)* | S82407 | F | ACCTTCACCTGCAACGTAGC | 148 | 0.97 |
| | | R | GGGTGTCTTTGGGTTTCGGT | | |
| Immunoglobulin heavy constant gamma 3 *(IGHG3)* | U63638 | F | GCTCAGAAACCCAGACCTTCA | 232 | 0.95 |
| | | R | TCCTTGGGTTTCGGTGGGAA | | |
| Immunoglobulin heavy constant mu 1 *(IGHM1)* | U63637 | F | CCCAGGACTTCATGCCCAAT | 278 | 1.08 |
| | | R | CGGGACAAAGACACTCACGA | | |
| Immunoglobulin heavy constant mu 2 *(IGHM2)* | AY230207 | F | CTTTCAAGGGCCGGATGACT | 130 | 1.08 |
| | | R | CGGGACAAAGACACTCACGA | | |
| C-C motif chemokine ligand 28 *(CCL28)* | NM_001101163.1 | F | GCTGACGGGGATTGTGACTT | 257 | 1.02 |
| | | R | CACTCCTCTGTGCAGCTTCATC | | |
| C-C motif chemokine receptor 10 *(CCR10)* | NM_001194964.1 | F | GTCTCGCCCTGCTATTGGAT | 139 | 1.06 |
| | | R | TAGAGCACTGGATTGAGGCC | | |
| Mucosal vascular addressin cell adhesion molecule 1 *(MADCAM1)* | NM_001037821.1 | F | GAGATCCACCAGTCGTCAGC | 265 | 1.05 |
| | | R | ACATCGTCGGGGAGCAGATTT | | |
| Polymeric immunoglobulin receptor *(PIGR)* | NM_174143.1 | F | TGCACATTACCAGCCTGAGG | 129 | 0.93 |
| | | R | GGATTGCCGTCTCTTCATTGAC | | |
| Fc gamma receptor and transporter *(FCGRT)* | NM_176657.1 | F | CCAAGTTTGCCCTGAACG | 161 | 1.09 |
| | | R | GTGTGGGCAGGAGTAGAGGA | | |
| Activation induced cytidine deaminase *(AICDA)* | NM_001038682.3 | F | GGAGTCCAGATCGCCATC | 172 | 0.92 |
| | | R | GCAAGTCATCAACCTCGTAG | | |

*Efficiency = 10(-1/slope), the slope was calculated from a standard curve constructed using a dilution series of pooled cDNA.
