# Supplementary material for: Developmental changes in the capacity for mucosal immunoglobulin production and secretion in the intestines of growing calves
Source: Vet Res. 2025 Nov 19;56:220. doi: 10.1186/s13567-025-01648-z (PMC12628562; doi:10.1186/s13567-025-01648-z)
Supplement: Supplementary file 3 — Additional file 3. Antibodies used for immunohistochemistry. [file 13567_2025_1648_MOESM3_ESM.pdf]

Antibody name	Antibody class	Host animal	Product information
Anti-bovine IgA	IgG	Sheep	Fortis life science, A10-131F
Anti-bovine IgG	IgG	Sheep	Fortis life science, A10-118F
Anti-bovine IgM*	IgG	Sheep	Fortis life science, A10-101A
Normal sheep IgG*	IgG	Sheep	Sigma Aldrich, I5131

*Anti-bovine IgM antibody and normal sheep IgG were manually conjugated with FITC using a commercial kit (Dojindo, LK-01)
