# Supplementary figures and images for: Developmental changes in the capacity for mucosal immunoglobulin production and secretion in the intestines of growing calves
Source: Vet Res. 2025 Nov 19;56:220. doi: 10.1186/s13567-025-01648-z (PMC12628562; doi:10.1186/s13567-025-01648-z)

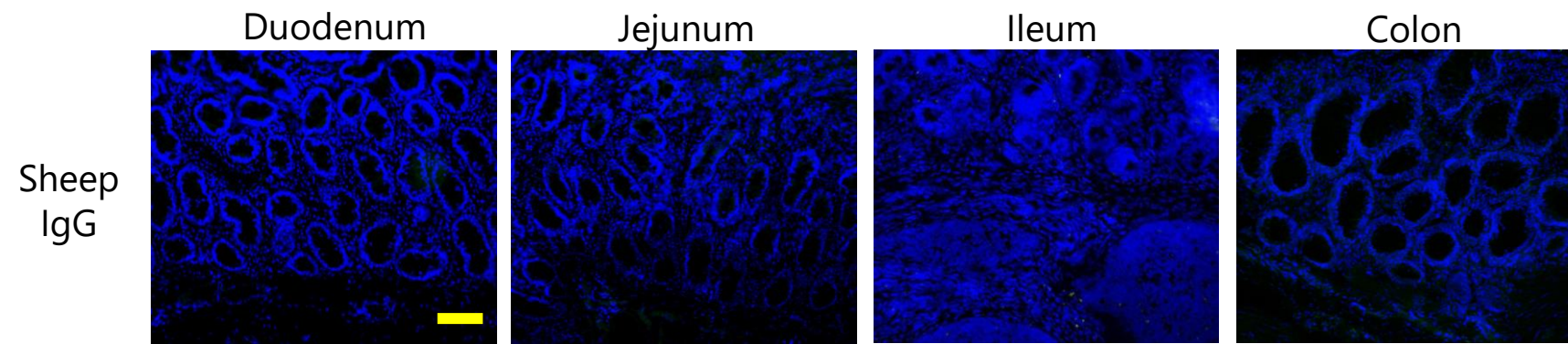

Supplement: Supplementary file 4 — Additional file 4. Isotype control of immunohistochemistry of intestinal tissues from calves. Intestinal sections from calves at 12 weeks of age were reacted with normal sheep IgG conjugated with FITC (n = 4: representative pictures are shown). Nuclei were counterstained (blue). The yellow scale bar indicates 100 μm. [file 13567_2025_1648_MOESM4_ESM.pdf]
