# Supplementary material for: Developmental changes in the capacity for mucosal immunoglobulin production and secretion in the intestines of growing calves
Source: Vet Res. 2025 Nov 19;56:220. doi: 10.1186/s13567-025-01648-z (PMC12628562; doi:10.1186/s13567-025-01648-z)
Supplement: Supplementary file 5 — Additional file 5. Descriptive statistics for the data corresponding to Figure 2. [file 13567_2025_1648_MOESM5_ESM.pdf]

Site	Gene	mean	sd	max	min
Duodenum	*IGHA*	0.987527	1.341801	1.973323	-0.54054
	IGHG	-0.47045	0.041912	-0.43018	-0.51383
	IGHM	-0.51708	0.044297	-0.46676	-0.5502
Jejunum	*IGHA*	1.14459	1.024195	2.327225	0.550587
	IGHG	-0.54979	0.040155	-0.51973	-0.59539
	IGHM	-0.5948	0.014044	-0.57881	-0.60512
Ileum	*IGHA*	0.810649	1.490395	2.507792	-0.28502
	IGHG	-0.23815	0.428386	0.25646	-0.49121
	IGHM	-0.5725	0.181046	-0.37131	-0.7223
Colon	*IGHA*	1.04653	1.197418	1.902776	-0.32177
	IGHG	-0.40295	0.241555	-0.15246	-0.63444
	IGHM	-0.64358	0.012664	-0.63552	-0.65817
Duodenum	*IGHG1*	1.166319	0.968341	2.106831	0.172363
	IGHG2	-0.57188	0.013675	-0.55722	-0.58428
	IGHG3	-0.59444	0.032713	-0.55801	-0.6213
Jejunum	*IGHG1*	1.112523	1.101079	1.939759	-0.13724
	IGHG2	-0.54697	0.031458	-0.52423	-0.58287
	IGHG3	-0.56555	0.039212	-0.52412	-0.60209
Ileum	*IGHG1*	1.182328	0.921104	2.244696	0.606818
	IGHG2	-0.58126	0.074734	-0.5033	-0.65228
	IGHG3	-0.60107	0.020283	-0.58097	-0.62153
Colon	*IGHG1*	0.953784	1.397038	2.405332	-0.38148
	IGHG2	-0.4676	0.02948	-0.43966	-0.49841
	IGHG3	-0.48618	0.017933	-0.47449	-0.50683
Duodenum	*IGHM1*	0.046628	1.270075	1.506114	-0.80768
	IGHM2	-0.04663	0.938289	0.99071	-0.8361
Jejunum	*IGHM1*	0.173483	1.46796	1.868537	-0.67441
	IGHM2	-0.17348	0.504781	0.284275	-0.71485
Ileum	*IGHM1*	0.612958	1.097913	1.868255	-0.16827
	IGHM2	-0.61296	0.409187	-0.23531	-1.04769
Colon	*IGHM1*	-0.22585	0.914344	0.457941	-1.26442
	IGHM2	0.225854	1.229205	0.972261	-1.19286
