# Supplementary material for: Developmental changes in the capacity for mucosal immunoglobulin production and secretion in the intestines of growing calves
Source: Vet Res. 2025 Nov 19;56:220. doi: 10.1186/s13567-025-01648-z (PMC12628562; doi:10.1186/s13567-025-01648-z)
Supplement: Supplementary file 6 — Additional file 6. Descriptive statistics for the data corresponding to Figure 3. [file 13567_2025_1648_MOESM6_ESM.pdf]

Gene	Site	mean	sd	max	min
IGHA	Duodenum	0.374241	1.314281	1.339818	-1.12249
	Jejunum	0.622925	1.022952	1.804125	0.029643
	Ileum	-0.94329	0.150876	-0.77149	-1.05421
	Colon	-0.05388	0.756493	0.487075	-0.91833
IGHG1	Duodenum	-0.21516	0.513141	0.283229	-0.74188
	Jejunum	-0.40806	0.48379	-0.04459	-0.95717
	Ileum	-0.16826	0.502101	0.410845	-0.48198
	Colon	0.79148	1.8572	2.721144	-0.98359
IGHG2	Duodenum	-0.36517	0.241045	-0.10665	-0.58375
	Jejunum	-0.39625	0.459763	-0.06382	-0.92094
	Ileum	-0.14351	1.355095	1.270082	-1.43134
	Colon	0.904925	1.303635	2.140486	-0.45751
IGHG3	Duodenum	-0.48905	0.939335	0.556977	-1.2605
	Jejunum	-0.33436	0.933588	0.652012	-1.20423
	Ileum	-0.06556	0.599113	0.528205	-0.66988
	Colon	0.888974	1.291827	1.731503	-0.59832
IGHM1	Duodenum	0.074583	1.475346	1.769953	-0.9178
	Jejunum	-0.34085	0.64099	0.399301	-0.71109
	Ileum	0.946874	0.805852	1.868243	0.373467
	Colon	-0.68061	0.201949	-0.52958	-0.90999
IGHM2	Duodenum	0.398258	1.877315	2.473749	-1.1813
	Jejunum	-0.39165	0.379644	-0.04738	-0.79881
	Ileum	0.537455	0.517302	1.01488	-0.01214
	Colon	-0.54406	0.467619	-0.26011	-1.08377
