# Supplementary material for: Developmental changes in the capacity for mucosal immunoglobulin production and secretion in the intestines of growing calves
Source: Vet Res. 2025 Nov 19;56:220. doi: 10.1186/s13567-025-01648-z (PMC12628562; doi:10.1186/s13567-025-01648-z)
Supplement: Supplementary file 7 — Additional file 7. Descriptive statistics for the data corresponding to Figure 5. [file 13567_2025_1648_MOESM7_ESM.pdf]

Gene	Site	Age	mean	sd	max	min
	Duodenum	04w	-0.61261	0.14107	-0.40808	-0.71974
	Duodenum	13w	-0.07976	0.735458	0.748799	-0.72978
	Duodenum	40w	0.923157	1.455049	1.992155	-0.73388
	Jejunum	04w	-0.7227	0.043701	-0.66603	-0.77136
	Jejunum	13w	-0.13156	0.684884	0.729715	-0.70559
	Jejunum	40w	1.139013	1.12619	2.439421	0.485856
IGHA	Ileum	04w	-0.69983	0.078968	-0.62628	-0.77173
	Ileum	13w	0.375211	1.285557	2.220944	-0.73437
	Ileum	40w	0.432831	0.984654	1.554076	-0.29104
	Colon	04w	-0.62665	0.082104	-0.51286	-0.70603
	Colon	13w	-0.25405	0.362652	0.267776	-0.57016
	Colon	40w	1.174274	1.345274	2.136249	-0.36299
	Duodenum	04w	-0.58231	0.10405	-0.42701	-0.64752
	Duodenum	13w	-0.41797	0.24136	-0.14296	-0.64787
	Duodenum	40w	1.333698	1.095765	2.397972	0.208946
	Jejunum	04w	-0.81676	0.080047	-0.72896	-0.91458
	Jejunum	13w	0.104364	0.735097	1.129478	-0.42915
	Jejunum	40w	0.949863	1.21264	1.860915	-0.42652
IGHG1	Ileum	04w	-0.85315	0.175451	-0.67029	-1.07
	Ileum	13w	0.244213	0.961405	1.427241	-0.92398
	Ileum	40w	0.811909	1.001792	1.967338	0.185985
	Colon	04w	-0.48688	0.091318	-0.36065	-0.56998
	Colon	13w	-0.27854	0.289745	0.12997	-0.51495
	Colon	40w	1.020555	1.634013	2.718324	-0.5412
	Duodenum	04w	-0.69994	0.159145	-0.46662	-0.81982
	Duodenum	13w	-0.36987	0.504765	0.33079	-0.79608
	Duodenum	40w	1.426407	0.523872	1.988255	0.951351
	Jejunum	04w	-0.83406	0.168969	-0.65688	-1.04469
	Jejunum	13w	0.174369	1.021087	1.549187	-0.63253
	Jejunum	40w	0.879583	0.883536	1.518417	-0.12871
IGHG2	Ileum	04w	-0.62507	0.222112	-0.32554	-0.86039
	Ileum	13w	0.221529	0.832837	1.296761	-0.73649
	Ileum	40w	0.538048	1.603672	2.210945	-0.98602
	Colon	04w	-0.4751	0.195972	-0.19163	-0.63966
	Colon	13w	-0.4892	0.165567	-0.25959	-0.6467
	Colon	40w	1.285736	1.221279	2.443241	0.009374

IGHG3	Duodenum	04w	-0.44205	0.042126	-0.38091	-0.4726
	Duodenum	13w	-0.46566	0.006446	-0.45625	-0.47057
	Duodenum	40w	1.210281	1.405563	2.77549	0.055927
	Jejunum	04w	-0.49588	0.033198	-0.45164	-0.52679
	Jejunum	13w	-0.447	0.144625	-0.23021	-0.52671
	Jejunum	40w	1.257176	1.305688	2.636686	0.040606
	Ileum	04w	-0.57224	0.233688	-0.22559	-0.72846
	Ileum	13w	-0.45301	0.507752	0.307832	-0.73633
	Ileum	40w	1.366992	0.814263	2.173992	0.545655
	Colon	04w	-0.49827	0.085002	-0.41466	-0.59722
	Colon	13w	-0.53492	0.126064	-0.34596	-0.60207
	Colon	40w	1.377592	1.024623	2.045851	0.19793
IGHM1	Duodenum	04w	-0.07249	0.568339	0.756659	-0.53128
	Duodenum	13w	-0.34118	0.46295	0.074029	-0.76062
	Duodenum	40w	0.551567	1.869316	2.69966	-0.70582
	Jejunum	04w	-0.06907	0.83058	1.03591	-0.79804
	Jejunum	13w	-0.19818	0.907188	1.156786	-0.71595
	Jejunum	40w	0.35634	1.565937	2.164528	-0.54815
	Ileum	04w	-0.44836	0.590417	0.397565	-0.8926
	Ileum	13w	-0.47604	0.491959	0.231401	-0.90743
	Ileum	40w	1.232539	0.989978	2.364429	0.528117
	Colon	04w	0.501764	1.220682	1.986015	-0.57936
	Colon	13w	-0.45789	0.466134	-0.0815	-1.09508
	Colon	40w	-0.0585	1.229223	0.860779	-1.45472
IGHM2	Duodenum	04w	0.028056	0.74468	1.083989	-0.64915
	Duodenum	13w	-0.38621	0.65269	0.296292	-0.95267
	Duodenum	40w	0.477537	1.699182	2.356091	-0.95214
	Jejunum	04w	0.412491	0.535176	0.945124	-0.14869
	Jejunum	13w	-0.30076	1.500666	1.811521	-1.62924
	Jejunum	40w	-0.14897	0.798673	0.575299	-1.00553
	Ileum	04w	-0.47388	0.684811	0.39146	-1.1044
	Ileum	13w	-0.37864	0.848077	0.782047	-1.04793
	Ileum	40w	1.136702	0.737354	1.817216	0.353313
	Colon	04w	0.284898	0.642339	1.0734	-0.47496
	Colon	13w	-0.75734	0.388569	-0.19621	-1.07606
	Colon	40w	0.629928	1.499978	1.540755	-1.10131
