# Supplementary material for: Developmental changes in the capacity for mucosal immunoglobulin production and secretion in the intestines of growing calves
Source: Vet Res. 2025 Nov 19;56:220. doi: 10.1186/s13567-025-01648-z (PMC12628562; doi:10.1186/s13567-025-01648-z)
Supplement: Supplementary file 8 — Additional file 8. Descriptive statistics for the data corresponding to Figure 6. [file 13567_2025_1648_MOESM8_ESM.pdf]

Gene	Site	Age	mean	sd	max	min
CCL28	Duodenum	04w	0.096644	0.775993	0.918788	-0.60055
	Duodenum	13w	-0.10319	0.928744	0.803195	-1.2771
	Duodenum	40w	0.008729	1.662171	1.875629	-1.3105
	Jejunum	04w	0.167105	1.030489	1.244652	-0.94666
	Jejunum	13w	0.219547	1.353394	1.366589	-1.71646
	Jejunum	40w	-0.51554	0.32971	-0.21872	-0.87043
	Ileum	04w	0.056188	1.27953	1.847454	-1.11623
	Ileum	13w	0.559093	0.673001	1.006717	-0.43771
	Ileum	40w	-0.82037	0.473076	-0.5062	-1.36447
	Colon	04w	0.573492	0.447285	1.075485	-0.01336
	Colon	13w	0.086041	0.797568	1.083856	-0.56025
	Colon	40w	-0.87938	1.383179	0.505329	-2.26102
CCR10	Duodenum	04w	-0.31463	0.713362	0.752094	-0.7371
	Duodenum	13w	0.255263	1.273863	1.965356	-1.01004
	Duodenum	40w	0.079151	1.210325	0.969543	-1.29894
	Jejunum	04w	-0.09386	0.930863	1.1919	-1.03195
	Jejunum	13w	0.099255	1.557917	2.148872	-1.62194
	Jejunum	40w	-0.0072	0.148946	0.15705	-0.1335
	Ileum	04w	-0.08905	0.868122	0.831332	-1.2156
	Ileum	13w	-0.27787	0.856512	0.533882	-1.21572
	Ileum	40w	0.489224	1.496608	2.211065	-0.4993
	Colon	04w	0.149111	1.049951	1.661297	-0.64648
	Colon	13w	-0.30222	1.173661	1.429718	-1.11181
	Colon	40w	0.204145	0.995252	1.264483	-0.70981
PIGR	Duodenum	04w	-0.6091	0.176826	-0.37371	-0.76783
	Duodenum	13w	-0.17881	0.558596	0.288876	-0.98847
	Duodenum	40w	1.050553	1.422534	2.538427	-0.29611
	Jejunum	04w	-0.7862	0.19109	-0.53474	-0.99668
	Jejunum	13w	0.128893	1.19096	1.649722	-1.06732
	Jejunum	40w	0.876406	0.629345	1.403381	0.179562
	Ileum	04w	-0.56793	0.163425	-0.33193	-0.70886
	Ileum	13w	0.718504	1.4347	2.650178	-0.55074
	Ileum	40w	-0.20076	0.366527	0.173488	-0.55904
	Colon	04w	-0.7727	0.720331	-0.03097	-1.43641
	Colon	13w	0.258231	0.834626	1.295666	-0.70357
	Colon	40w	0.685963	1.069327	1.645631	-0.46673

FCGRT	Duodenum	04w	0.769317	0.705582	1.343272	-0.13496
	Duodenum	13w	-0.37666	1.090139	1.249502	-1.03446
	Duodenum	40w	-0.52354	0.769443	0.129607	-1.37174
	Jejunum	04w	0.676655	1.521191	2.928026	-0.28728
	Jejunum	13w	-0.39965	0.235951	-0.07349	-0.57316
	Jejunum	40w	-0.36934	0.075328	-0.28518	-0.43045
	Ileum	04w	0.42558	0.889542	1.602805	-0.55837
	Ileum	13w	0.40869	0.900309	1.592556	-0.56843
	Ileum	40w	-1.11236	0.211969	-0.93177	-1.34573
	Colon	04w	0.363295	0.590732	1.093613	-0.18328
	Colon	13w	-0.05787	1.145098	0.954231	-1.44648
	Colon	40w	-0.40723	1.410766	1.2058	-1.41087
AICDA	Duodenum	04w	-0.63886	0.592856	0.242064	-0.99115
	Duodenum	13w	0.564596	1.376824	2.374496	-0.94857
	Duodenum	40w	0.099018	0.400982	0.403958	-0.35519
	Jejunum	04w	-0.21522	1.095325	1.166892	-1.1341
	Jejunum	13w	0.085256	0.426843	0.651522	-0.32561
	Jejunum	40w	0.173284	1.665798	2.05689	-1.10602
	Ileum	04w	-0.51552	0.23812	-0.2242	-0.73813
	Ileum	13w	-0.59077	0.21822	-0.31223	-0.76738
	Ileum	40w	1.475059	0.591835	2.150912	1.049465
	Colon	04w	-0.52652	0.049187	-0.4867	-0.59835
	Colon	13w	0.415105	1.58147	2.786149	-0.44507
	Colon	40w	0.148551	0.559132	0.652762	-0.45278
MADCAM1	Duodenum	04w	0.008394	0.632294	0.947129	-0.42805
	Duodenum	13w	-0.11207	1.301153	1.499165	-1.4412
	Duodenum	40w	0.138229	1.344205	1.072886	-1.40227
	Jejunum	04w	0.070096	1.186835	1.597201	-1.11947
	Jejunum	13w	-0.03755	1.353745	1.924863	-0.99061
	Jejunum	40w	-0.0434	0.35032	0.359292	-0.27799
	Ileum	04w	-0.66439	0.526891	0.001083	-1.1047
	Ileum	13w	0.21211	1.216041	1.645415	-1.00072
	Ileum	40w	0.603043	0.920402	1.647629	-0.08887
	Colon	04w	0.080092	1.122649	1.555421	-1.15618
	Colon	13w	-0.47812	0.729651	0.556833	-1.08376
	Colon	40w	0.530698	1.190976	1.568568	-0.76961
