# Supplementary material for: Developmental changes in the capacity for mucosal immunoglobulin production and secretion in the intestines of growing calves
Source: Vet Res. 2025 Nov 19;56:220. doi: 10.1186/s13567-025-01648-z (PMC12628562; doi:10.1186/s13567-025-01648-z)
Supplement: Supplementary file 9 — Additional file 9. Descriptive statistics for the data corresponding to Figure 7. [file 13567_2025_1648_MOESM9_ESM.docx]

| Gene | Site | mean | sd | max | min |
| --- | --- | --- | --- | --- | --- |
| *CCL28* | Duodenum | 0.722933 | 1.76321 | 2.703316 | -0.67649 |
|  | Jejunum | 0.209331 | 0.218816 | 0.406315 | -0.0262 |
|  | Ileum | -0.76992 | 0.072653 | -0.72167 | -0.85348 |
|  | Colon | -0.16234 | 0.748031 | 0.586517 | -0.90954 |
| *CCR10* | Duodenum | 0.105269 | 1.849764 | 1.466075 | -2.00089 |
|  | Jejunum | 0.141372 | 0.173927 | 0.33317 | -0.00611 |
|  | Ileum | -0.43908 | 1.153076 | 0.887532 | -1.2007 |
|  | Colon | 0.192436 | 0.572017 | 0.801861 | -0.33285 |
| *MADCAM1* | Duodenum | -0.64207 | 0.67986 | -0.16934 | -1.42121 |
|  | Jejunum | -0.68279 | 0.255769 | -0.38878 | -0.85406 |
|  | Ileum | 0.411186 | 1.030092 | 1.580261 | -0.36318 |
|  | Colon | 0.913669 | 1.042983 | 1.822571 | -0.22506 |
| *PIGR* | Duodenum | 0.976315 | 1.469246 | 2.513047 | -0.41457 |
|  | Jejunum | 0.117589 | 0.323538 | 0.3885 | -0.24065 |
|  | Ileum | -1.03639 | 0.046058 | -0.98936 | -1.08141 |
|  | Colon | -0.05752 | 0.409727 | 0.310193 | -0.49919 |
| *AICDA* | Duodenum | -0.60638 | 0.010682 | -0.59825 | -0.61847 |
|  | Jejunum | -0.54268 | 0.099179 | -0.43053 | -0.61885 |
|  | Ileum | 1.604831 | 0.556319 | 2.240126 | 1.204777 |
|  | Colon | -0.45578 | 0.111565 | -0.35517 | -0.57576 |
| *FCGRT* | Duodenum | 0.061577 | 1.016427 | 0.924378 | -1.05889 |
|  | Jejunum | 0.007131 | 0.313906 | 0.357836 | -0.24754 |
|  | Ileum | -0.89988 | 0.383426 | -0.57321 | -1.32202 |
|  | Colon | 0.831173 | 1.401644 | 2.433776 | -0.16597 |
